# Multi-site screening for *Pneumocystis jirovecii* in lung cancer: possible tumour tissue colonization

**DOI:** 10.3389/fcimb.2026.1755638

**Published:** 2026-03-04

**Authors:** Magdalena Szydłowicz, Żaneta Zajączkowska, Mariusz Chabowski, Maciej Nowicki, Błażej Łukianowski, Pawel Gajdzis, Martin Kváč, Enrique J. Calderón, Solène Le Gal, Marta Kicia

**Affiliations:** 1 Department of Biology and Medical Parasitology, Wroclaw Medical University, Wroclaw, Poland; 2 Department of Surgery, 4th Military Clinical Hospital, Wroclaw, Poland; 3 Department of Clinical Surgical Sciences, Faculty of Medicine, Wroclaw University of Science and Technology, Wroclaw, Poland; 4 Department of Pathomorphology, 4th Military Clinical Hospital, Wroclaw, Poland; 5 Department of Clinical Pathology, Wroclaw Medical University, Wroclaw, Poland; 6 Institute of Parasitology, Biology Centre of the Academy of Sciences of the Czech Republic, České Budějovice, Czechia; 7 Faculty of Agriculture and Technology, University of South Bohemia, České Budějovice, Czechia; 8 Instituto de Biomedicina de Sevilla/Hospital Universitario Virgen del Rocío/Consejo Superior de Investigaciones Científicas/Universidad de Sevilla, Seville, Spain; 9 Centro de Investigación Biomédica en Red de Epidemiología y Salud Pública, Madrid, Spain; 10 Departamento de Medicina, Hospital Universitario Virgen del Rocío, Facultad de Medicina, Universidad de Sevilla, Seville, Spain; 11 Laboratory of Parasitology and Mycology, Brest University Hospital, Brest, France; 12 Fungal Respiratory Infections (FRI) Research Unit, University of Angers, University of Brest, Brest, France

**Keywords:** genotyping, histological malignancy grade, lung neoplasms, normal adjacent tissue, *Pneumocystis* colonization

## Abstract

**Objectives:**

Recent studies suggest that various tumour types can be colonized by different microorganisms, but data on unusual opportunistic fungus – *Pneumocystis jirovecii –* remain scarce. Lung cancer patients are considered one of the risk groups for its infection. Since *P. jirovecii* tends to distribute focally within the lungs, this study aimed to determine whether it can be detected in lung tumour tissue.

**Methods:**

Fragments of neoplastic tissue (NPL), normal adjacent tissue (NAT) and respiratory secretions (RS) were collected from 70 patients with histologically confirmed primary lung cancer. DNA was extracted and analysed by nested-PCR targeting the *mtLSU* rRNA and *CYB* loci, followed by genotyping.

**Results:**

*Pneumocystis jirovecii* was detected in fourteen samples derived from 8/70 individuals (11.4%): two NPL, six NAT and six RS. In two patients, *Pneumocystis* was detected in all three specimen types; both were diagnosed with the same histological malignancy grade (G3, *P*=0.036). The genotype distribution varied across sample types in most cases.

**Conclusions:**

The ability of *Pneumocystis* to colonize NPL may be linked to the stage of tumour advancement, suggesting that local tumour-related factors could influence its colonization. These findings support further investigation of the lung microbiome in the context of tumour-associated microenvironments and their potential utility as complementary biomarkers in lung cancer.

## 1 Introduction

Pathogens such as bacteria, viruses and fungi can induce the host immune system when they colonize different tissues. It may be hypothesized that pro-inflammatory immune responses triggered by infection by certain species may contribute to local tissue changes that, over time, may promote carcinogenesis in the affected organ (Kostic et al., 2013). Conversely, cancer progression itself might create an altered microenvironment that facilitates colonization by specific pathogens (Wang et al., 2023). Although these possibilities require further investigation, developing knowledge about this relationship may have the potential to be reflected in practical medical applications, for instance, facilitating the diagnosis of early-stage cancer, predicting the risk of metastasis using co-infecting pathogens as specific biomarkers, or implementing the early diagnosis and rapid elimination of infectious agents as a preventive measure (Riquelme et al., 2019; Dohlman et al., 2022).

Recent studies, focused on the analysis of the mycobiomes in cancer tissues of various types, have shown correlations between the frequency of specific fungal species and the type of cancer diagnosed, with the *Blastomyces* genus found to be most abundant in the case of lung cancer (Dohlman et al., 2022). Moreover, infection with an atypical respiratory fungus, *Pneumocystis jirovecii*, is also frequently observed in patients with lung cancers (Mori et al., 2010; Sokulska et al., 2018). The diagnosis of this pathogen increasingly relies on PCR, however, due to high sensitivity of this method, detection of *Pneumocystis* DNA in respiratory samples does not necessarily indicate active infection (Oz and Hughes, 1999; Medrano et al., 2005), but may instead reflect colonization, which is clinically relevant due to the potential risk of subsequent development of *Pneumocystis* pneumonia (PCP) in colonized individuals. Independent risk factors for colonization include long-term use of high doses of steroids and concurrent chemoradiotherapy, as well as lymphopenia and coexisting pulmonary diseases, while advanced lung cancer and a weakened general condition of the patient further increase the poor prognosis. The causal relationship between *Pneumocystis* and lung cancer is not clear – general weakening of the immune system as a side effect of oncological treatment may predispose to colonization by this opportunistic fungus, which is characterised by pulmonary tropism. Moreover, it has been shown for most advanced cancers that the tumour itself may also cause a state of immunosuppression, which further predisposes to opportunistic infections, including *Pneumocystis* (Kiessling et al., 1999; De La Horra et al., 2004). Specific types of cancer may be more closely correlated with the occurrence of *Pneumocystis*, since they might create specific conditions that facilitate its colonization (De La Horra et al., 2004). On the other hand, *Pneumocystis* colonization has been shown to correlate with an increase in the level of inflammatory markers and mucins – the main component of mucus, a barrier protecting the respiratory tract against chemical, physical and biological factors, the decomposition of which is characteristic of various chronic respiratory diseases (Calderón et al., 2007; Pérez et al., 2014; Rojas et al., 2019; Rojas et al., 2023). Indeed, it was suggested that inflammatory process and surfactant changes induced by even a low number of these microorganisms at very early stages of infection may activate or facilitate the development of chronic obstructive pulmonary disease (Varela et al., 2003; Calderón et al., 2007), therefore its potential role in cancer progression cannot be excluded. However, little is known about the likelihood of *Pneumocystis* to settle within lung tumours (De La Horra et al., 2004). Since a tendency for focal concentration of *Pneumocystis* in the lungs has been demonstrated (Vargas et al., 2017), it is conceivable that such accumulation may also occur in neoplastic lung tissue.

In the absence of suitable *in vitro* culture systems for *Pneumocystis*, molecular approach based on sequence typing involving the analysis of single nucleotide polymorphisms (SNPs) distribution across multiple genetic loci is currently considered the preferred strategy for epidemiological studies on *Pneumocystis* transmission and diversity (Beard et al., 2004). It provides high discriminatory power and allows for the detection of mixed infections with multiple *P. jirovecii* isolates within a single patient, a relatively common phenomenon (Maitte et al., 2013; Pasic et al., 2020).

Among the various genetic targets commonly used in *Pneumocystis* genotyping, multi-copy mitochondrial genes are particularly advantageous, enabling amplification even at very low fungal burdens. The mitochondrial large subunit ribosomal RNA (*mtLSU* rRNA) gene encodes a component essential for mitochondrial translation and represents the primary molecular target used worldwide for *Pneumocystis* detection (Beard et al., 2000; Valero et al., 2016). Owing to its relatively high variability among isolates, *mtLSU* rRNA is regarded as a highly informative marker for studies on genetic diversity (Alanio et al., 2016; Pasic et al., 2020). Cytochrome b (*CYB*) is another multi-copy gene widely applied in molecular epidemiological studies, although it exhibits slightly lower variability than *mtLSU* rRNA (Pasic et al., 2020). It has been shown that the SNP profile may correlate with clinical data of the colonized individual (Esteves et al., 2010a; Sokulska et al., 2018), as well as with specific properties of the infecting organism (Esteves et al., 2011). For instance, our previous studies in a group of patients with various respiratory diseases demonstrated statistically significant differences in the SNP pattern within the *CYB* gene between individuals diagnosed with lung cancer and other study participants (Sokulska et al., 2018).

To date, the colonization of lung cancer tissue by *P. jirovecii* has not been well documented. Since the predisposition to localise in specific areas of the lungs of the same host may also be associated with the presence of particular SNPs in the *Pneumocystis* genome (Ambrose et al., 2001), the aim of the present study was to investigate whether *P. jirovecii* can be detected in the lung tumour tissue and to examine the potential relationship of such focusing with the SNPs present in the *mtLSU* rRNA and *CYB* genes of the colonizing strain.

## 2 Materials and methods

### 2.1 Patients and specimens

The study included patients hospitalised due to suspected lung cancer at the Department of Surgery of the 4th Military Clinical Hospital (Wroclaw, Poland) between July 2023 and June 2025. Patients were eligible for inclusion if they were over 18 years of age, provided informed consent to participate in the study, had a diagnosis of primary lung cancer confirmed by histopathological examination, and had not received chemotherapy or immunosuppressive treatment for six months prior to enrolment. All patients underwent positron emission tomography/computed tomography (PET/CT) for tumour imaging as part of the routine preoperative assessment. During resection of a suspected cancer lesion, respiratory secretions (RS; bronchial secretion from the lower respiratory tract, taken by an anaesthesiologist through an intubation tube during an operative procedure), and fragments of lung tissue – both neoplastic (NPL) and those showing no visible pathological changes (normal adjacent tissue, NAT) – were collected intraoperatively and aseptically from each patient. Samples were frozen at −20 °C without preservatives (RS) or placed in RNAlater™ (Thermo Fisher Scientific, Carlsbad, CA, United States) stabilisation solution (NPL and NAT) before molecular examination. In addition, to evaluate samples for the presence of *Pneumocystis* developmental forms, histopathological examination of lung tissue was performed using standard staining procedures, including Giemsa and Grocott–Gomori methenamine silver (GMS) staining, and indirect immunofluorescence assays (MonoFluo *P. jirovecii* IFA Test Kit; Bio-Rad) were applied to respiratory secretions (RS). Demographic, clinical and laboratory data were recorded for all individuals included in the study. Informed consent, approved by the Human Research Ethics Committee of Wroclaw Medical University according to agreement no. KB-143/2023, was obtained from all study participants. The datasets analysed in this study have been deposited in the secured Polish Platform of Medical Research of the Wroclaw Medical University [https://ppm.umw.edu.pl/info/researchdata/UMW9db8fd8e71a24a34bd02591badeb9258/], DOI: 10.60956/x08c-a904.

### 2.2 *Pneumocystis jirovecii* detection and SNP identification

Lung tissue samples (NPL and NAT) weighing 20–25 mg each, as well as respiratory secretion sediments (obtained after treatment with dithiothreitol as a mucolytic agent) were subjected to mechanical homogenisation by bead disruption (Precellys, Bertin Technologies, France) and proteinase K digestion (1–3 hours, 56 °C). Total DNA was extracted from each sample using a DNeasy^®^ Blood & Tissue Kit (Qiagen, Hilden, Germany) according to the manufacturer’s protocol, with an additional step of phenol:chloroform:isoamyl alcohol pre-treatment (Pé́rez-Brocal et al., 2020). Nested polymerase chain reaction (PCR) with a Taq polymerase and primers targeting a partial sequence of the *P. jirovecii mtLSU* rRNA gene was performed to detect *P. jirovecii* DNA in patients’ samples according to the protocol described previously (Wakefield, 1996). Human-derived *Pneumocystis* DNA sample and molecular grade water were used as positive and negative controls in each experiment, respectively. The molecular examination was repeated three times for each sample, always accompanied by a negative control (molecular grade water). Agarose gel electrophoresis was used to visualise of PCR products, and bands corresponding to 260 bp (expected size for *mtLSU* rRNA amplicon) were purified with a Zymoclean Gel DNA Recovery Kit (Zymo Research, Irvine, CA, USA). DNA was sequenced bi-directionally by a company offering this service commercially, enabling confirmation of *Pneumocystis* detection, as well as identification of SNPs and corresponding *mtLSU* rRNA genotypes (Beard et al., 2000).

The nested-PCR protocol for amplification of the *CYB* locus was also applied for all *mtLSU* rRNA-positive samples (Monroy-vaca et al., 2014; Esteves et al., 2010b). Bands of the expected size (620 bp) visualised by electrophoresis were processed in the same way as described above, in order to perform phylogenetic analyses based on the identification of SNPs at informative positions, according to previously described nomenclature (Esteves et al., 2010b).

The nucleotide sequences were analysed with ChromasPro 2.1.4 (Technelysium, Pty, Ltd., South Brisbane, Australia) and aligned to reference sequences from GenBank using BioEdit v.7.0.5 (Hall, 1999). The phylogenetic relationships between the *P. jirovecii* strains detected in this study and other *Pneumocystis* isolates were analysed using the Neighbour-Joining method with 1000 bootstrap replicates and the best-fitting DNA substitution model, both selected in MEGA11. The phylograms were processed with CorelDRAW X7. The sequences were deposited in GenBank under the Accession Numbers (Acc. nos.): PV928554–PV928567 for *CYB* and PV918862–PV918875 for *mtLSU* rRNA.

### 2.3 Statistical analysis

Categorical variables were compared between patients and sample types using Fisher’s exact test, and continuous variables using the Mann-Whitney U test, with a *P* value < 0.05 considered significant.

## 3 Results

Primary lung cancer was confirmed in 70 study participants by a histopathological examination. The majority of diagnoses (64/70, 91.4%) corresponded to non-small cell lung carcinomas – NSCLC (invasive non-mucinous adenocarcinoma, large cell carcinoma, squamous cell carcinoma, adenosquamous carcinoma and pleomorphic carcinoma), while the remaining diagnoses included small cell lung carcinomas – SCLC (2/70, 2.8%), as well as typical (3/70, 4.3%) and atypical (1/70, 1.4%) carcinoids (Detterbeck, 2018; Nicholson et al., 2022). The median age of all tested patients (n=70, including 39 males and 31 females) was 68 (range 44–82) years. All samples for *Pneumocystis* examination were collected intraoperatively during resection of pulmonary lesions from the right (n=32) or left (n=38) lung (**Table 1**).

**Table 1:** Basic characteristics of individuals with confirmed colonization and *Pneumocystis* genotypes identified in different types of specimens.

Patient no.	Diagnosis	Histological grade^a^	*Pneumocystis jirovecii* genotypes detected in tested samples					
			NPL		NAT		RS	
			*mtLSU* rRNA	*CYB*	*mtLSU* rRNA	*CYB*	*mtLSU* rRNA	*CYB*
1	adenocarcinoma	3	2	2	2	2	2	2
2	adenocarcinoma	3	1 + 2^b^	5 + 8^b^	2	5	1	5
3	SCLC	1	n/d	n/d	1	8	1	1
4	adenocarcinoma	2	n/d	n/d	2	1	3	1
5	adenocarcinoma	2	n/d	n/d	n/d	n/d	2	1
6	adenocarcinoma	2	n/d	n/d	n/d	n/d	2	8
7	squamous cell carcinoma	1	n/d	n/d	3	1	n/d	n/d
8	adenocarcinoma	2	n/d	n/d	2	8	n/d	n/d

NPL, fragment of neoplastic tissue; NAT, normal adjacent tissue; RS, respiratory secretions; *mtLSU* rRNA, mitochondrial large subunit rRNA; *CYB*, cytochrome b; SCLC, small cell lung carcinoma; n/d, *Pneumocystis* DNA was not detected in this sample.

a according to Moreira et al (Moreira et al., 2020).

b a mixture of two divergent genotypes detected in one sample.

**Figure 1:**
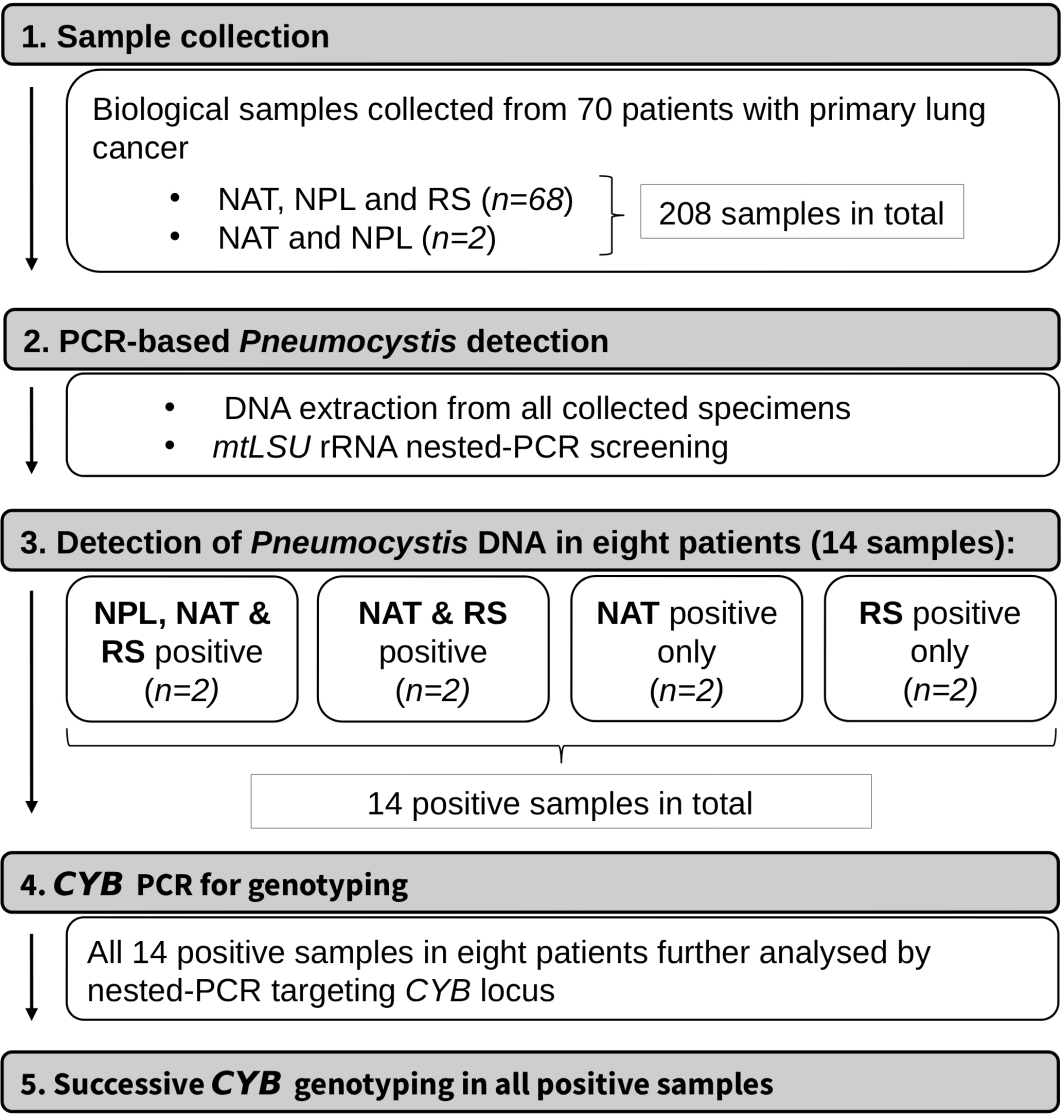
Diagnostic and genotyping workflow strategy. NAT, normal adjacent tissue; NPL, fragment of neoplastic tissue; RS, respiratory specimen; *mtLSU* rRNA, mitochondrial large subunit rRNA; *CYB*, cytochrome b.

The diagnostic and genotyping workflow strategy of the present study is illustrated in **Figure 1**. RS were not collected from two study participants for technical reasons, while all three types of samples were obtained from the remaining 68 patients. In total, *P. jirovecii* DNA was detected in 14 samples from 8 patients (8/70, 11.4%). These included NPL samples from two individuals (2/8, 25%): a 67-year-old woman (patient no. 1) and a 68-year-old man (patient no. 2), diagnosed with tumours in the left and right lung, respectively. Both patients had the same diagnosis (grade 3 invasive non-mucinous adenocarcinoma), which was not shared by any other *Pneumocystis*-positive patient. In each of these two cases, the presence of *Pneumocystis* was also confirmed in NAT and RS. In addition, *P. jirovecii-*specific DNA was detected in both NAT and RS samples in two study participants (patients nos. 3 and 4), in RS alone in another two (patients nos. 5 and 6), and in NAT alone in two others (patients nos. 7 and 8, **Table 1**). Histopathological examination using standard staining procedures and indirect immunofluorescence assays did not allow for the direct detection of *Pneumocystis* forms in patients’ samples. Imaging studies revealed no radiological findings suggestive of PCP, and none of the patients exhibited typical clinical features of pneumocystosis (such as progressive dyspnoea, non-productive cough, low-grade fever).

**Figure 2:**
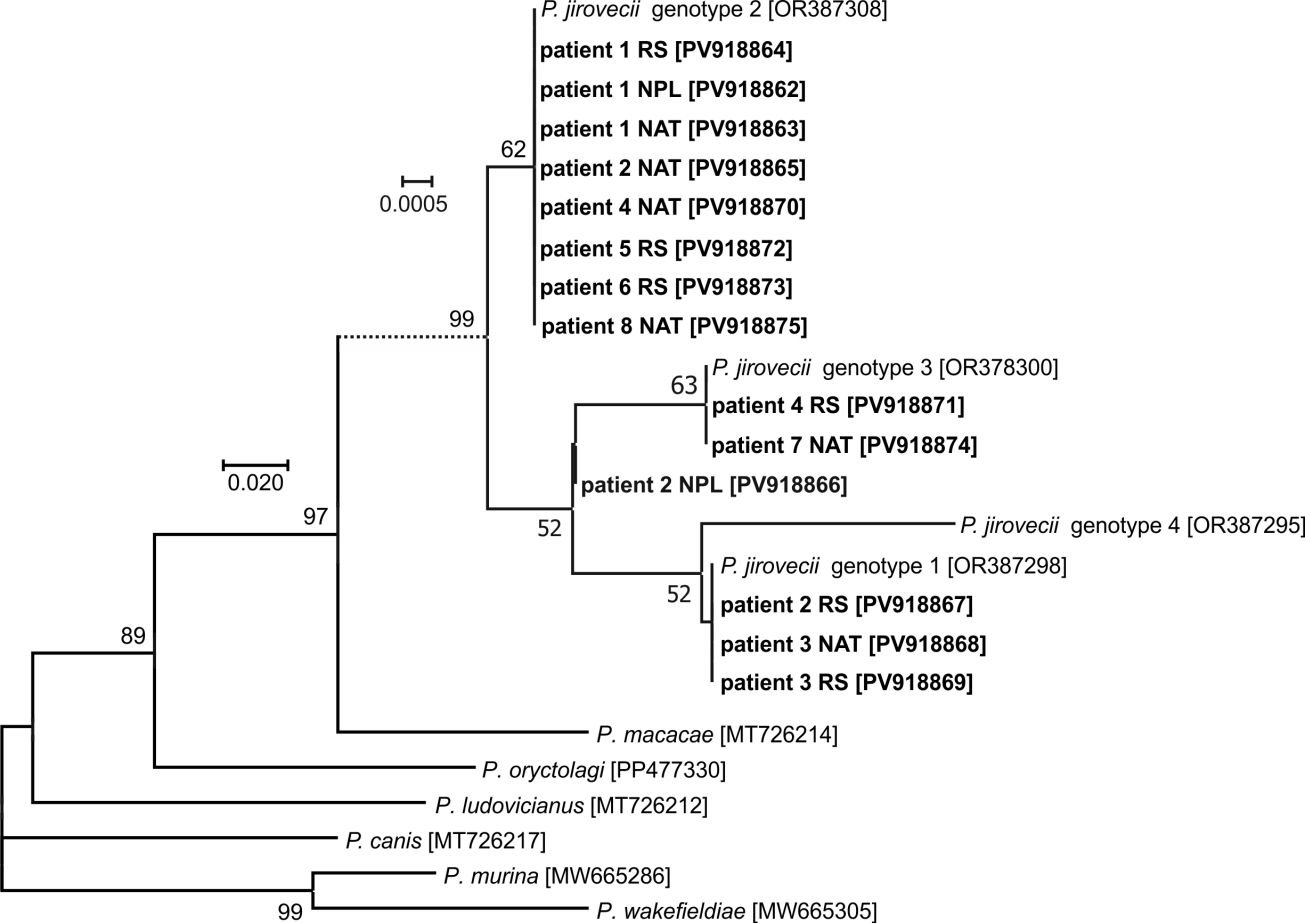
Phylogenetic tree of the *mtLSU* rRNA region of *Pneumocystis jirovecii* isolates detected in this study. Tamura’s 3-parameter model was applied, using a discrete Gamma distribution. Bootstrap values with more than 50% bootstrap support are shown at the nodes. The branch length scale bar, indicating the number of substitutions per site, is included. The sequences obtained in this study are shown in bold and labelled with the patient number and type of biological material (RS, respiratory specimen; NAT, normal adjacent tissue; NPL, fragment of neoplastic tissue). The GenBank accession number is given in brackets.

**Figure 3:**
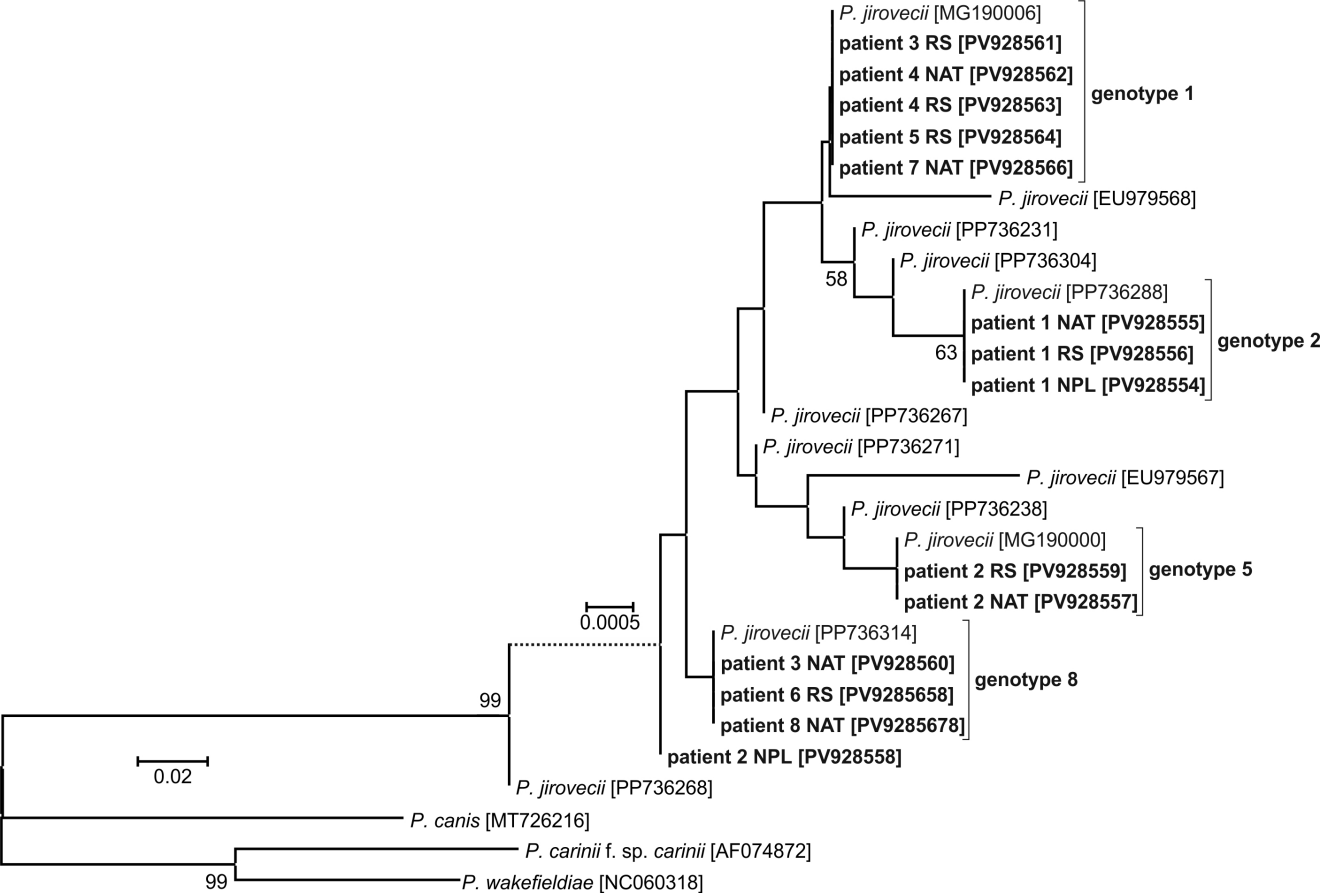
Phylogenetic tree of the *CYB* region of *Pneumocystis jirovecii* isolates detected in this study. Tamura’s 3-parameter model was applied, using a discrete Gamma distribution. Bootstrap values with more than 50% bootstrap support are shown at the nodes. The branch length scale bar, indicating the number of substitutions per site, is included. The sequences obtained in this study are shown in bold and labelled with the patient number and type of biological material (RS, respiratory specimen; NAT, normal adjacent tissue; NPL, fragment of neoplastic tissue). The GenBank accession number is given in brackets.

Analysis of SNPs in the *mtLSU* rRNA locus in all *Pneumocystis*-positive samples revealed that genotype 2 was predominant (detected in 8/14 samples, 57.1%), while genotypes 1 and 3 were detected in three (21.4%) and two (14.3%) samples, respectively; in one case, a mixture of genotypes 1 and 2 was observed (**Figure 2**). For *CYB*, genotype 1 was observed most frequently (five cases, 35.7%), genotypes 2 and 8 occurred in three samples each (21.4%), genotype 5 in two (14.3%), and a mixture of genotypes 5 and 8 was detected in one case (**Figure 3**).

In patient no. 1, the genotype distribution was identical in all three samples – for both *mtLSU* rRNA and *CYB*, genotype 2 was observed in NPL, NAT and RS alike. In the remaining patients, the detected *CYB* or *mtLSU* rRNA genotypes only partially overlapped (**Table 1**). In patient no. 2, a mixture of two divergent genotypes was detected in the NPL sample for both *mtLSU* rRNA and *CYB*. Corresponding NAT and RS collected from this individual presented *mtLSU* rRNA genotypes 2 and 1, respectively, while *CYB* genotype 5 was observed in both of them.

A comparison of the most important clinical, demographic and laboratory data between the *Pneumocystis*-positive and *-*negative patients is shown in **Table 2**. No statistically significant differences were found between these two groups, but the detection of *Pneumocystis* in the NPL was limited to patients diagnosed with grade 3 lung adenocarcinoma, indicating a potential positive correlation between high-grade tumour and NPL fungal colonization (*P*=0.036, Fisher Exact Test).

**Table 2:** Comparison of clinical and demographic characteristics of individuals with (Positive) and without (Negative) *Pneumocystis jirovecii* colonization confirmed in at least one type of collected specimen.

Characteristic	*Pneumocystis jirovecii* result		*P* value
	Positive (n=8)	Negative (n=62)	
Age in years, median (range)	68 (66–76)	69 (44–82)	0.542
Sex			
Male	4 (50)	35 (56.5)	0.726
Female	4 (50)	27 (43.5)	
Lung affected			
Right	3 (37.5)	29 (46.8)	0.718
Left	5 (62.5)	33 (53.2)	
Diagnosis			
NSCLC	7 (87.5)	57 (91.9)	0.531
SCLC	1 (12.5)	1 (1.6)	0.217
others	0	4 (6.4)	1.000
Histological Grade^a^			
1	2 (25)	12 (19.3)	0.669
2	4 (50)	22 (35.4)	0.701
3	2 (25)	25 (40.3)	0.459
Lung cancer stage^b^			
I	5 (62.5)	46 (74.2)	0.669
II	1 (12.5)	6 (9.7)	0.556
III	0	5 (8.1)	1.000
IV	1 (12.5)	3 (4.8)	0.364
Pleural invasion^c^			
PL0	4 (50)	36 (58)	0.418
PL1	3 (37.5)	18 (29)	0.674
PL2	1 (12.5)	1 (1.6)	0.398
PL3	0	1 (1.6)	1.000
Necrosis within the tumour			
present	4 (50)	21 (33.9)	1.000
absent	4 (50)	20 (32.2)	
data not available	0	21 (33.9)	
Lymphovascular invasion	6 (75)	29 (46.8)	0.260
Perineural invasion	1 (12.5)	2 (3.2)	0.317
Comorbidities			
COPD	4 (50)	15 (24.2)	0.199
arterial hypertension	2 (25)	29 (46.8)	0.287
other respiratory diseases^d^	3 (37.5)	12 (19.4)	0.355
previous cancer diseases^e^	1	17 (27.4)	0.670
coronary artery disease	0	14 (22.6)	0.343
diabetes	0	9 (14.5)	0.584
a history of heart attack	0	3 (4.8)	1.000
epilepsy	0	1 (1.6)	1.000
Blood gas analysis			
pO_2_ [mmHg], median (range)	70.6 (57–77.3)	70 (47.7–106)	0.849
pCO_2_ [mmHg], median (range)	38.2 (34.8–45)	38.5 (4.2–46.9)	0.667
pH, median (range)	7.42 (7.0–7.47)	7.41 (7.35–7.46)	0.787
Spirometry			
FVC in litres, median (range)	2.61 (1.6–4.91)	3.05 (1.05–4.88)	1.000
predicted FVC value [%], median (range)	75 (65–128)	91 (41–137)	0.865
FEV1 in litres, median (range)	1.76 (0.81–2.9)	1.85 (0.6–3.97)	0.896
predicted FEV1 value [%], median (range)	65 (30–119)	72 (32–116)	0.447
FEV1/FVC, median (range)	0.57 (0.36–0.78)	0.65 (0.31–0.86)	0.624
Cigarette smoking	6 (75)	51 (82.3)	0.636
Number of pack years, median (range)	40 (25–100)	40 (7–80)	0.638

Data represent number (%) unless otherwise indicated. SCLC, small cell lung carcinoma; NSCLC, non-small cell lung carcinoma; COPD, chronic obstructive pulmonary disease; FVC, forced vital capacity; FEV1, forced expiratory volume in one second.

a According to Moreira et al (Moreira et al., 2020); data for three patients were not available.

b According to American Joint Commission on Cancer (AJCC) (Detterbeck, 2018); data for three patients were not available.

c PL0, no pleural invasion; PL1, tumour invasion beyond the elastic layer of the visceral pleura; PL2, tumour invasion to the visceral pleural surface; PL3, tumour invasion of the parietal pleura (according to AJCC) (Detterbeck, 2018); data for six patients were not available.

d including emphysema, chronic bronchitis and post-tuberculosis changes in the lungs.

e including history of prostate, large intestine, breast, kidney, bladder, stomach, duodenum, uterus and tonsil cancer.

## 4 Discussion

*Pneumocystis* is an opportunistic pathogen of the respiratory tract. One of the risk groups for infection is patients with lung cancer. Since it has been shown that this fungus tends to concentrate as clusters within human lungs (Vargas et al., 2017), the aim of this study was to investigate whether it has the ability to colonize lung tumour tissue. As a result, presence of *P. jirovecii* DNA was observed within the lung neoplastic tissue in two of 70 patients (2.8%), which accounted for 25% of all *Pneumocystis*-positive individuals. These cases confirm the ability of the fungus to colonize tumour tissue, but it does not seem to be its preferential site, particularly since in both patients with *Pneumocystis*-positive NPL, the fungal DNA was also found in corresponding NAT and RS. Moreover, in the remaining six patients with confirmed colonization, *Pneumocystis* was observed in NAT and/or RS, but not in the NPL. Although the identification of the pathogen within the NAT does not confirm the presence of *Pneumocystis* in the tumour itself, the colonization in its vicinity may also be important in the context of focalization in a given area of ​​the lungs, potentially associated with local tissue tumour-dependent changes.

Previous studies have shown associations between specific pathogens and clinical parameters of patients with lung cancer, including early cancer detection, patient survival, and treatment effectiveness (Cheng et al., 2024; Narunsky-Haziza et al., 2022). Accordingly, analyses of the micro- and mycobiota have been proposed as potential biomarkers in cancers diagnosis and prognosis (Dong et al., 2021; Dohlman et al., 2022; Dohlman et al., 2021; Yan et al., 2015). Increasing evidence also highlights the critical role of the tumour microenvironment (TME) in cancer progression (Liu et al., 2026; Son et al., 2017; Edirisinghe et al., 2025). The lung cancer TME is highly heterogeneous and characterised by altered immune cell infiltration and extensive stromal interactions that collectively promote tumour growth, invasion and immune evasion. Notably, several non-cancerous cellular components of TME have been shown to establish an immunosuppressive niche that inhibits effective anti-tumour immune responses and facilitates tumour progression in NSCLC (Edirisinghe et al., 2025).

Contemporary cancer treatment strategies increasingly extend beyond targeting tumour cells alone to include modulation of the TME components and their interactions with the tumour (Witz, 2009). Recent studies emphasise the dynamic interplay between hypoxia, metabolic reprogramming, and immune regulation in shaping the lung TME and contributing to therapeutic resistance (Liu et al., 2026). Moreover, investigations correlating tumour grade with immune infiltrates have found that higher-grade tumours may exhibit elevated numbers of tumour-associated macrophages (TAMs) and cancer-associated fibroblasts (CAFs), which support tumour growth, enhance resistance to therapy (Li et al., 2018; Ohtaki et al., 2010) and promote cancer cell invasion (Neri et al., 2015), thereby contributing to aggressive clinical behaviour and poorer outcomes (Kubota et al., 2024). Chronic inflammation within TME may further drive l ocal immunosuppression, facilitating not only tumour progression, but also the potential colonization of opportunistic pathogens (Tan et al., 2021). Of note, the only patients with the fungus identified within the NPL in the present study, were also the only ones diagnosed with grade 3 lung adenocarcinoma in the *Pneumocystis-*positive group. Grade 3 is the highest histological malignancy grade which indicates the lowest degree of differentiation and is characterised by high malignancy potential, usually associated with a worse prognosis (Moreira et al., 2020). This observation may suggest a potential association between the stage of cancer advancement and *Pneumocystis* colonization of neoplastic tissue.

In this context, it can be hypothesized that high-grade tumours may create a microenvironment permissive to opportunistic colonization: decreased local immune surveillance and altered stromal architecture may impair antifungal defence within the tumour milieu, while chronic inflammation and stromal remodelling promote pathogen persistence (Narunsky-Haziza et al., 2022). Since no significant difference in Grade 3 diagnosis frequency was observed between *Pneumocystis-*positive and -negative individuals in the present study (*P* > 0.05), this tumour grade does not appear to be associated with a higher risk of colonization. However, in colonized individuals with Grade 3 tumours, a tendency towards NPL may be suggested, although this observation should be interpreted with caution given that it is based on only two *Pneumocystis*-positive cases. Taken together, these data support the conclusion that the neoplastic lung tissue fragment is not a preferential location for the pathogen, however, it cannot be ruled out that *Pneumocystis* colonization may reflect underlying immunological and inflammatory states within the lung tumour microenvironment, particularly in higher-grade malignancies. Although the present findings are preliminary, they support the concept that analyses of lung micro- and mycobiota may have future potential as complementary biomarkers, aiding in the characterization of tumour-associated microenvironments and contributing to disease monitoring in lung cancer.

Detection of *Pneumocystis* DNA by PCR is a highly sensitive approach; however, it does not provide direct evidence of viable, intact infectious organisms within the host (Medrano et al., 2005). Confirmation of active infection requires meeting additional diagnostic criteria, including microscopic visualisation of cysts directly in respiratory specimens or quantitative molecular assessment of the pathogen burden (Brown et al., 2024). Nevertheless, in the absence of clinical symptoms and imaging findings suggestive of PCP, the patients in the current study were classified as colonized, a condition that carries important epidemiological implications as well. *Pneumocystis* colonization, even though asymptomatic, is an important factor due to the possibility of inducing an inflammatory response and potentially contributing to lung tissue damage (Calderón et al., 2007). Furthermore, colonized patients with lung cancer may be at risk of PCP symptoms developing further due to weakened immunity as a side effect of oncological treatment (Alanio and Bretagne, 2017; Mori et al., 2009). Among patients diagnosed with lung cancer, the presence of *Pneumocystis* has been shown to be significantly higher in SCLC patients compared to NSCLC (Shan et al., 2025). Similarly, in the study by De La Horra et al., 2004, conducted on lung tissue sections collected post*-*mortem from individuals diagnosed with SCLC and NSCLC, *Pneumocystis* colonization was demonstrated in all cases from the first group (including neoplastic tissue), but only 20% in the latter. This prevalence is much higher compared to the present study, but it is noteworthy that the vast majority of patients included in the current report was diagnosed with NSCLC – the most common type of lung cancer (Molina et al., 2008). Moreover, the two cohorts differ substantially in the clinical context: the patients in the previous study were examined following the full course of disease, while the samples in the present study were collected at the time of diagnosis, prior to the initiation of oncological treatment. The present study’s participants did not exhibit any PCP symptoms nor clinical signs of infection and had not yet undergone chemotherapy, which is a PCP risk factor. The observation of fungal DNA is therefore most likely related to asymptomatic colonization, which is associated with a low fungal burden; hence, in some cases, it was detected only in one type of collected samples. Additionally, the *Pneumocystis* screening in the current study was limited to fragments of tissues resected during surgery, while in the aforementioned report the analyses were conducted post-mortem, therefore it allowed for more extensive sampling from multiple lung regions, potentially increasing the probability of identifying the pathogen. The type of material being tested has a great impact on the detection sensitivity. On the one hand, the examination of lung tissue is a more invasive and sensitive method than the analysis of respiratory secretions; however, due to the heterogenous distribution of the fungus in the lungs (Helweg-Larsen et al., 2001; Vargas et al., 2017; Ambrose et al., 2001), it is likely to result in negative detection when it is performed on a fragment not occupied by the clusters of the pathogen.

Among the loci typically used in *Pneumocystis* genotyping, in the present study we selected *mtLSU* rRNA and *CYB* – two mitochondrial multi-copy genes, allowing reliable amplification even in case of a very low level of colonization. By applying this approach, genotypic variants can be analysed in greater depth, facilitating assessment of potential differences in SNP composition between lung regions. Importantly, discordant genotypes observed between the *mtLSU* rRNA and *CYB* loci within the same sample should not be interpreted as evidence of colonization with a mixture of different strains, but may rather reflect independent evolutionary dynamics of mitochondrial loci or the presence of mixed mitochondrial haplotypes. The overall distribution of *mtLSU* rRNA and *CYB* genotypes was comparable to our previous findings in a group of patients with various respiratory diseases (Sokulska et al., 2018). This suggests that the genotype distribution is likely not related to the underlying diagnosis or specific part of the lung colonized, but rather reflects the incidence of the given strains in a designated geographic area. Interestingly, in one of the NPL-positive patients, both *CYB* and *mtLSU* rRNA genotyping were uniform across all three sample types. In contrast, divergent genotypes were detected in different sample types from other patients in whom *Pneumocystis* was detected in more than one specimen type. In the second NPL-positive individual, a mixture of genotypes was observed specifically in the neoplastic tissue. It has been shown in studies of genotypic diversity that, in most cases, active PCP involves a complex mixture of strains and genotypes (Alanio et al., 2016), likely reflecting ongoing exposure to *Pneumocystis* throughout life (Gigliotti and Wright, 2012). The relative proportions of particular genotypes may fluctuate during colonization, influenced by factors such as drug use and comorbidities. Diversity may also arise from mutations acquired during colonization (Alanio et al., 2016), particularly in mitochondrial loci, where point mutations occur more frequently than in the nuclear genome due to higher replication rates, exposure to reactive oxygen species, and less efficient DNA repair mechanisms (Song et al., 2005). Additionally, differences between specimen types may have an impact, as *P. jirovecii* genotypes can vary between isolates from respiratory secretions and lung tissue, aligning with previous reports on intrapulmonary compartmentalization (Helweg-Larsen et al., 2001; Ambrose et al., 2001). Finally, the infection intensity affects the detectability of genotype mixtures; in cases of low fungal burden, as observed in colonization in our study, minor genotypes may be missed due to the dominance of a single strain (Alanio et al., 2016). Of note, *CYB* genotypes 2 and 5 were each detected in only one patient, and in both cases, these were individuals with a *Pneumocystis-*positive NPL specimen. Further studies in larger cohorts are needed to explore whether strains with specific genotypes could exhibit a preference for colonizing tumour tissue.

This study has several limitations. The relatively small sample size, and consequently the low proportion of NPL-positive cases, mean that a potential association with the degree of tumour differentiation should be interpreted with caution. While this observation is of interest in the present study group, further, more comprehensive studies are required to verify it, involving larger patient cohorts and additional diagnostic methods that allow direct visualization of *P. jirovecii* in the tested material, since PCR-based diagnostics, while highly sensitive, do not allow for the detection of viable organisms in the tested material. Furthermore, this study includes patients at an early stage of diagnosis and therefore does not account for the effects of treatments administered later in the disease course. The initiation of anticancer therapy, which generally predisposes patients to opportunistic infections, may influence the distribution of colonization across different lung regions and potentially enhance certain predispositions. Continued investigation in future studies is therefore warranted.

To the best of our knowledge, this is the first study analysing *P. jirovecii* directly in NPL, NAT and RS from the same patient. Our findings suggest that this pathogen may act not only as a secondary colonizer but also as a microorganism whose presence and distribution could be potentially shaped by features of the tumour microenvironment, albeit this possibility remains to be further explored. Although the colonization of the lung neoplastic tissue by *Pneumocystis* does not seem to be preferential, there may be a correlation between such tendency and the stage of tumour advancement. These results constitute a basis for future studies on the importance of the lung microbiome in the context of lung tumour immunology and potential biomarkers of cancer. The observed discrepancies in pathogen detection across different types of biological sample types indicate that a multi-specimen approach may be essential for a comprehensive assessment of *Pneumocystis* colonization. Finally, our results support the notion that lung cancer itself may be a risk factor for *Pneumocystis* colonization. Therefore, the implementation of routine screening in this risk group may be epidemiologically justified – particularly before the initiation of oncological treatment, which may increase the susceptibility to opportunistic infections.

## Data Availability

The datasets presented in this study can be found in online repositories. The names of the repository/repositories and accession number(s) can be found in the article/supplementary material.
